# Management of cancer pain with analgetic adjuvant and weak opioid in prostate cancer bone metastases: A case series

**DOI:** 10.1016/j.amsu.2020.10.070

**Published:** 2020-11-09

**Authors:** Jufriady Ismy, Dessy Rakhmawati Emril, Rizkidawati

**Affiliations:** a Urology Division, Surgery Department, Faculty of Medicine, Universitas Syiah Kuala, Zainoel Abidin General Hospital, Banda Aceh, Indonesia; b Pain and Headache Division, Neurology Department, Faculty of Medicine, Universitas Syiah Kuala, Zainoel Abidin General Hospital, Banda Aceh, Indonesia

**Keywords:** Cancer pain, Bone pain, Bone metastasis, Prostate cancer, Adjuvant therapy, Weak opioid

## Abstract

**Background:**

In cancer patients, cancer pain is the most common cancer complication. About 60–90% of patients with advanced stage cancer experience various levels of pain, and about 30% of patients have been suffering from persistent severe pain. Bones are the most frequent targets of metastases in patients with cancer such as breast, prostate, lung, kidney, and thyroid. In advanced prostate cancer, bone metastasis leads to bone pain, skeletal fracture, and increased mortality. At least 75% of patients with bone metastasis experience bone pain.

**Case description:**

We report three cases of cancer pain, treated with primary cancer from the prostate metastasis to the spine. All three patients had lower back pain that radiated to the left and right limbs, with mixed pain and bone pain, where early hospital admission shows the Numeric Rating Scale (NRS) pain scale 9–10. Treated with administration of adjuvant therapy (Gabapentin) and weak opioids (injections of Tramadol) as well as injections of Metylprednisolone (for 3 days), the patient's pain scale was evaluated, and the average NRS obtained on days 2–4 was 5–6. On day 5–8, treatment continued with Gabapentin and Tramadol injections, and the pain scale (NRS) decreased to 2–3. All patients on the 8-9^th^ day of treatment also received Biphosphonates to reduce pain, bone damage, fracture risk, and blood calcium levels. Patients can be discharged with an oral Gabapentin prescription only.

**Conclusion:**

A pain scale (NRS) reduction of >50% is obtained from the initial pain scale in cancer pain patients treated using a combination of adjuvant therapy and weak opioids.

## Highlights

• NRS reduction of 50% achieved by combination therapy.
• Weak opioid, adjuvant therapy, and steroid was effective.
• Patients can be discharged with an oral Gabapentin prescription only.

## 1 Introduction

In cancer patients, cancer pain is the most common cancer complication. About 60–90% of patients with advanced stage cancer experience various levels of pain, and about 30% of patients have been suffering from persistent severe pain [1].

In cancer, pain caused by nociceptor activation is called nociceptive pain; whereas pain caused by disorders of the nervous system is called neuropathic pain. According to research by Lucchesi, in pain induced by cancer both mechanisms can be involved, be it inflammatory or neuropathic, because tumor growth is responsible for tissue damage and release some inflammatory mediators. In addition, cancer cells can also compress and grow in sensory nerves or damage target tissue with consequent neuropathic changes. Cancer pain is also defined as a type of mixed pain [2,3].

Prostate cancer is the most common urologic malignancy worldwide. It's incidence is increasing due to the availability of screening tools such as the PSA and improvement in life expectancy [4]. About 90% of men with advanced stage prostate cancer will develop bone metastases [5].

Bone pain is the type of pain most experienced by cancer patients, and the main reason for this type of pain is due to the cancer metastasis in bone and the invasion of surrounding soft tissue violations [6].

Kader et al. suggested that compared using opioid monotherapy, a combination of gabapentin and an opioid is more effective to relieve neuropathic pain in cancer patients, and could represent a first line treatment for neuropathic cancer pain [7]. Informed consent has been given by the patient to be reported in a case report. This paper has been reported in line with the PROCESS criteria [8].

## 2 Case illustrations

**Fig. 1:**
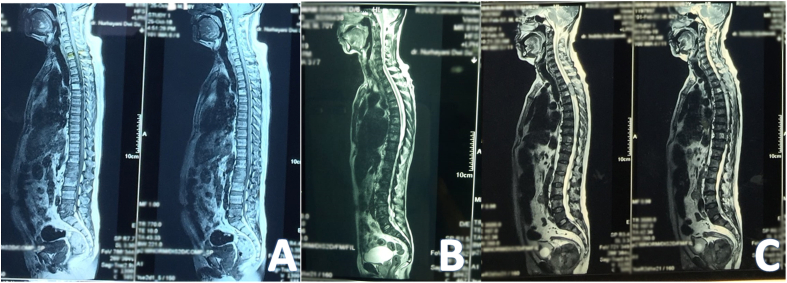
MRI wholespine; A. Case 1 (Mr.M), B. Case 2 (Mr.B), C. Case 3 (Mr.I).

This study is a case series with three consecutive patients. First case: Mr. M, 66 years old, complained of lower back pain that radiated to the left and right limbs for ±4 months, and worsted in the last month. The pain was described as sharp, throbbing, pressure, burning, and tingling. Pain disturbed sleep. Pain was also felt when the patient changed position from lying down, or from sitting to standing. In 2017 the patient underwent bilateral TUR-P with anatomic pathology: Adenocarsinoma prostate Gleason 3 + 3 = 6 and received Tapros therapy. Clinical examination: initial pain scale of 10/10 in severity on the Numeric Rating Scale. Pain DETECT: 16, DN4: 5/10. Motor strength 5 in all four extremities, lower back provocation tests were positive in both extremities, with limited range of motion (ROM) of spine due to pain. Routine hematological examination within normal limits, PSA Total: 1112.16 ng/ml. MRI of the spine showed a signal with pathological intensity in the thoracolumbosacral vertebral, a metastatic impression with cord compression (Fig. 1A). Patients treated with Tramadol injection 50 mg/8 h via syiringe pump were finished in 30 min, Metylprednisolone injection 125 mg/8 h and oral Gabapentin 300 mg–300 mg - 400 mg (Table 1, Table 2).

**Table 1:** Therapy of patients.

Day	Case 1	Case 2	Case 3
1	- IV methylprednisolone 125 mg/8 h - IV Tramadol 50 mg/8 h - Gabapentin 3 × 300 mg	- IV methylprednisolone 125 mg/8 h - IV Tramadol 50 mg/8 h - Gabapentin 3 × 300 mg	- IV methylprednisolone 125 mg/8 h - IV Tramadol 50 mg/8 h - Gabapentin 3 × 300 mg
2	- IV methylprednisolone 125 mg/8 h - IV Tramadol 50 mg/8 h - Gabapentin 300- 300–400 mg	- IV methylprednisolone 125 mg/8 h - IV Tramadol 50 mg/8 h - Gabapentin 3 × 300 mg	- IV methylprednisolone 125 mg/8 h - IV Tramadol 50 mg/8 h - Gabapentin 3 × 300 mg
3	- IV methylprednisolone 125 mg/8 h - IV Tramadol 50 mg/8 h - Gabapentin 300- 300–400 mg	- IV methylprednisolone 125 mg/12 h - IV Tramadol 50 mg/8 h - Gabapentin 3 × 300 mg	- IV methylprednisolone 125 mg/12 h - IV Tramadol 50 mg/8 h - Gabapentin 3 × 300 mg
4	- IV Tramadol 50 mg/8 h - Gabapentin 300- 300–400 mg	- IV Tramadol 50 mg/12 h - Gabapentin 3 × 300 mg	- IV Tramadol 50 mg/8 h - Gabapentin 3 × 300 mg
5	- IV Tramadol 50 mg/12 h - Gabapentin 300- 300–400 mg	- IV Tramadol 50 mg/12 h - Gabapentin 3 × 300 mg	- IV Tramadol 50 mg/12 h - Gabapentin 3 × 300 mg
6	- IV Tramadol 50 mg/12 h - Gabapentin 300- 300–400 mg	- IV Tramadol 50 mg/24 h - Gabapentin 3 × 300 mg	- IV Tramadol 50 mg/12 h - Gabapentin 3 × 300 mg
7	- IV Tramadol 50 mg/24 h - Gabapentin 300- 300–400 mg	- IV Tramadol 50 mg/24 h - Gabapentin 3 × 300 mg	- IV Tramadol 50 mg/24 h - Gabapentin 3 × 300 mg
8	- IV Tramadol 50 mg/24 h - Gabapentin 300- 300–400 mg	- Gabapentin 3 × 300 mg - Drip bondronate	- IV Tramadol 50 mg/24 h - Gabapentin 3 × 300 mg
9	- Gabapentin 300 - 300–400 mg - Drip bondronate	- Gabapentin 3 × 300 mg	- Gabapentin 3 × 300 mg - Drip bondronate
10	- Gabapentin 300 - 300–400 mg		- Gabapentin 3 × 300 mg

**Table 2:**
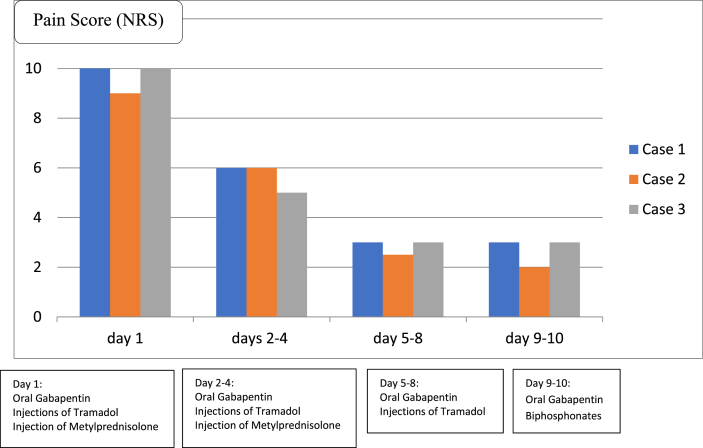
Follow up of patient's average pain score.

Second case: Mr. B, 71 years old, presented with low back pain that spread to both legs in the past ±6 months. Pain was described as burning, throbbing, aching, tingling and electric shock, which was worse at night. Patient had a history of prostate cancer 2 years ago, has undergone surgery with anatomic pathology: Adenocarsinoma prostate Gleason 3 + 3 = 6. Clinical examination: initial pain scale of 9/10 in severity on the Numeric Rating scale. PainDETECT: 15, DN4: 4/10. Myotom examination decreased in lower extremities with muscle strength 4. Mechanical tests were positive in both extremities, with limited ROM of spine due to pain. Routine hematological examination within normal limits, Total PSA: 1404.06 ng/ml. MRI of spine confirmed heterogenity of the signal intensity of the bone marrow C7, Th4, Th6, Th8, L3, with the impression of cervicothoracolumbar metastases with cord compression (Fig. 1B). The patient was treated with Tramadol injection 50 mg/8 h via a syrup pump for 30 min, Metylprednisolone injection 125 mg/8 h and oral Gabapentin 300 mg three times daily (Table 1).

Third case: Mr. I, age 61 years old, had complained about lower back pain for 2 months. Pain was described as numbness, tingling, throbbing, sharp with movement. Pain was accompanied by weakness of the lower limbs. Pain is continuous with severe intensity, especially at night. History of prostate cancer in 2019, has undergone surgery with anatomic pathology results: invasive Adeocarsinoma prostate gleason (4 + 3 = 7). Clinical examination: initial pain scale of 10/10 in severity on the Numeric Rating scale. PainDETECT: 14, DN4: 4/10. Myotom examination decreased in lower extremities with muscle strength 4, sensory examination within normal limits, movement test was positive in both extremities, with limited ROM of spine due to pain. Routine hematological examination within normal limits, total PSA: 904.66. MRI of spine demonstrated heterogeneity of signal intensity of bone marrow C1–C7, Th3, Th4, Th5, Th6, Th7, VL1-VL5, the impression of cervicothoracolumbal metastases with extradural SOL was as high as Th3–Th7 (Fig. 1C). Tramadol injection 50 mg/8 h via a syrup pump for 30 min, Metylprednisolone injection 125 mg/8 h and oral Gabapentin 300 mg three times daily (Table 1).

## 3 Discussion

We reported three cases of cancer pain, treated with primary cancer from the prostate metastasis to the spine. All three patients had lower back pain which radiated to the left and right limbs, with mixed pain and bone pain. Pain assessment of patients using the instrument PainDETECT and DN-4 obtained results of patients experiencing a component of neuropathic pain, nociceptive pain and central pain which is called mixed pain [3]. Patients also experienced bone pain which is characterized by increased pain if the patient moves/changes position. The pain scale assessment is evaluated based on the degree of pain; severe pain is evaluated hourly, moderate pain every 4 h, and mild pain every 8 h.

At initial hospital admission, all three patients entered with severe pain NRS 9–10, and were treated with administration of adjuvant therapy (Gabapentin) and weak opioids (injections of Tramadol) as well as injections of Metylprednisolone (for three days). In the first two days there was a 20% reduction in the pain scale. Metylprednisolone (steroids) are widely used for cancer-related pain including both neuropathic and bone pain [1,9]. Based on the European Society for Medical Oncology (ESMO), in bone pain with compression of the spinal cord, Metylprednisolone (steroids) are immediately given when there are clinical and radiological symptoms [10].

For a wide range of conditions from moderate to severe acute and chronic pain including in cancer pain, tramadol is used in the management of pain. Tramadol consists of two enantiomers and is a synthetic analog of codeine and morphine, both of which promote analgesic activity via different mechanisms. Tramadol undergoes CYP2D6 dependent O-methylation to demethyltramadol (M1). Tramadol inhibits norepinephrine reuptake and (+)-tramadol inhibits serotonin reuptake, thus, pain transmission in the spinal cord is greatly inhibited [11]. Research by Vijayan Et al. in 2018 about the use of Tramadol in Southeast Asian countries states that almost all respondents use Tramadol to treat moderate cancer pain. Tramadol was chosen because it's efficacy for both neuropathic and nociceptive pain, and because patients prefer it over low-dose strong opioids, and as a step two analgesic on the pain ladder of WHO [12].

Adjuvant Therapy (Gabapentin) has a mechanism at the voltage-activated calcium channels in the central nervous system (CNS) and is effective in a wide range of neuropathic or mixed pain syndromes. Adjuvant therapy (Gabapentin, Pregabaline) is highly recommended as a first line treatment for neuropathic cancer pain (recommendation IA) [10,13]. Gabapentin is effective given in range of doses from 1800 to 3600 mg daily, in divided doses. A combination of opioids or non-opioids with anticonvulsants, can be an alternative because combination pharmacotherapy could increase analgesic efficacy and has the potential to reduce the overall side effect profile if synergistic effects allow for dose reductions of combined drugs [10,14]. Other authors determined that for treatment of tumour-related cancer pain there remains uncertainty regarding the risk-benefit trade-off from combining adjuvants and opioids [15]. In these cases a pain scale (NRS) reduction of >50% is obtained from the initial pain scale in cancer pain patients treated using a combination of adjuvant therapy and weak opioids.

The use of anticonvulsant drugs as adjuvant therapy for pain management in bone metastasis has increased throughout the year. Although research of high-quality evidence on the effect of antidepressants in cancer pain is scarce, a systematic review considers that there is enough evidence to place an antidepressant as adjuvant analgesic, alone or in combination with opioid. Primarily if there is neuropathic pain or a combination of nociceptive and neuropathic pain [16]. A study by Tagami et al. also describe the effectivity of pregabalin for radiculopathy pain and dexamethasone/betamethasone for brain tumor or headache related by metastases [17]. This is in line with our finding, where in our case the combination of steroid, adjuvant in addition to weak opiod reduced the sensation of pain.

Before allowed to discharge, on the 8-9th day all the patients received biphosphonates therapy. Biphosphonates are used to reduce pain, bone damage, fracture risk, and blood calcium levels [1,10,18].

## 4 Conclusion

In our cases, combination of adjuvant therapy and weak opioid could achieve pain scale (NRS) reduction of >50% from the initial pain scale in cancer pain patients. After discharge, patient was also able taking only Gabapentin afterwards. However, long term use of this combination should be evaluated to assess the possibility recurrent pain and adverse events.

## Provenance and peer review

Not commissioned, externally peer reviewed.

## Ethical approval

This is a case series; therefore, it did not require ethical approval from ethics committee. However, we have got permission from the patients to publish his data.

## Funding

We affirm that we did not receive any financial assistance from government or any private organization.

## Author contribution

Jufriady Ismy contributes in the study concept or design, data collection, analysis and interpretation, oversight and leadership responsibility for the research activity planning and execution, including mentorship external to the core team. ORCHID-ID: https://orcid.org/0000-0002-4462-7433

Dessy Rakhmawati Emril contributes in the study concept or design, data collection, analysis and interpretation, oversight and leadership responsibility for the research activity planning and execution.

Rizkidawati contributes in the study concept or design, data collection, analysis and interpretation and writing the paper.

## Registration of research studies

We register the research at http://www.researchregistry.com.

UIN: researchregistry5959.

## Guarantor

Jufriady Ismy is the sole guarantor of this submitted article.

## Consent

Written informed consent was obtained from the patient for publication of this case report and accompanying images. A copy of the written consent is available for review by the Editor-in-Chief of this journal on request.

## Declaration of competing interest

We have no conflicts of interest to disclose.
